# Seasonal variation in the behaviour of a short-lived rodent

**DOI:** 10.1186/1472-6785-13-43

**Published:** 2013-11-15

**Authors:** Jana A Eccard, Antje Herde

**Affiliations:** 1 Animal Ecology, University of Potsdam, Maulbeerallee 1, 14469 Potsdam, Germany

**Keywords:** Animal personalities, Boldness, Life history, Pace-of-life, POL, Phenotypic plasticity, Common vole

## Abstract

**Background:**

Short lived, iteroparous animals in seasonal environments experience variable social and environmental conditions over their lifetime. Animals can be divided into those with a “young-of-the-year” life history (YY, reproducing and dying in the summer of birth) and an “overwinter” life history (OW, overwintering in a subadult state before reproducing next spring).

We investigated how behavioural patterns across the population were affected by season and sex, and whether variation in behaviour reflects the variation in life history patterns of each season. Applications of pace-of-life (POL) theory would suggest that long-lived OW animals are shyer in order to increase survival, and YY are bolder in order to increase reproduction. Therefore, we expected that in winter and spring samples, when only OW can be sampled, the animals should be shyer than in summer and autumn, when both OW and YY animals can be sampled.

We studied common vole (*Microtus arvalis*) populations, which express typical, intra-annual density fluctuation. We captured a total of 492 voles at different months over 3 years and examined boldness and activity level with two standardised behavioural experiments.

**Results:**

Behavioural variables of the two tests were correlated with each other. Boldness, measured as short latencies in both tests, was extremely high in spring compared to other seasons. Activity level was highest in spring and summer, and higher in males than in females.

**Conclusion:**

Being bold in laboratory tests may translate into higher risk-taking in nature by being more mobile while seeking out partners or valuable territories. Possible explanations include asset-protection, with OW animals being rather old with low residual reproductive value in spring. Therefore, OW may take higher risks during this season. Offspring born in spring encounter a lower population density and may have higher reproductive value than offspring of later cohorts. A constant connection between life history and animal personality, as suggested by the POL theory, however, was not found. Nevertheless, correlations of traits suggest the existence of animal personalities. In conclusion, complex patterns of population dynamics, seasonal variation in life histories, and variability of behaviour due to asset-protection may cause complex seasonal behavioural dynamics in a population.

## Background

Seasonal environments are characterized by variations in temperature, light conditions and nutrient or water availability throughout different seasons. Organisms have used a variety of strategies to adapt to seasonal environments; limiting reproduction to the most favourable time periods is one such example. For short-lived, iteroparous species with overlapping generations, individuals usually experience only one reproductive season during their lives, but several generations can reproduce during a single season. This pattern results in strong, annual population dynamics, as reported in zooplankton, insects or small mammals [1-4]. Seasonal variation also produces variation in the individual it affects the morphology of animals, including body size or fat layers [5,6], physiology such as the BMR (for review [7]), hormone secretion [8], and individual behaviour. Seasonal adaptations are possibly triggered by changes in photoperiod and mediated through neuroendocrinology [9].

**Figure 1:**
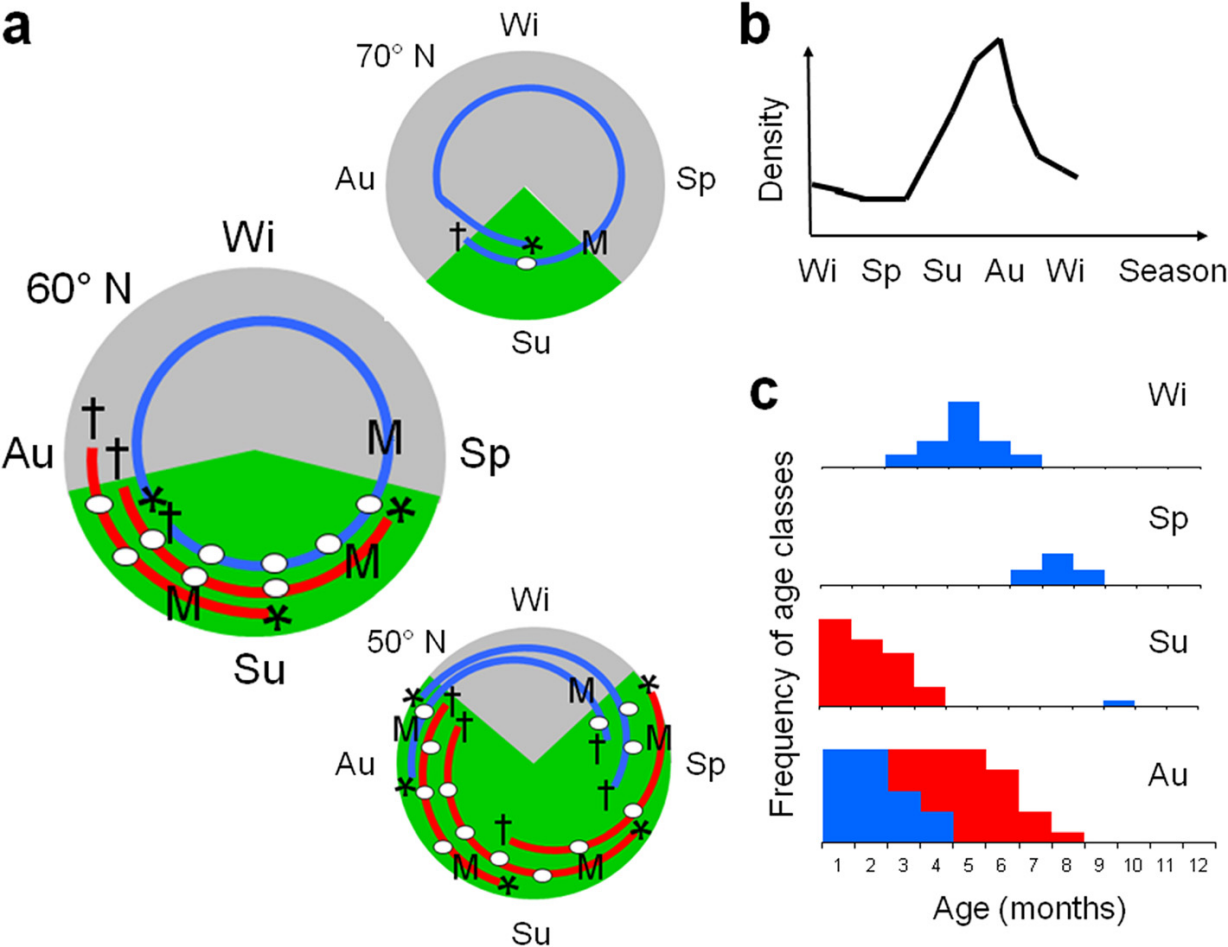
**Population dynamics and life history of short-lived, iteroparous, multivoltine animals in seasonal environments. (a)** Life history of small mammal cohorts around the year (winter (Wi), spring (Sp), summer (Su) and autumn (Au)) in different latitudes (after (Millar and Wille 1978)); dark grey area: non-reproductive winter season; green (light grey): reproductive summer season; blue lines (grey): overwinter (OW) animals, born in autumn; red lines (black): young-of-the year animals (YY), born in spring; * = birth, M = maturation, ┼ = death. **(b)** Typical, annual population dynamic; **(c)** age distribution per season. red (black): YY, blue (grey): OW.

Small mammals in seasonal environments also experience typical annual population fluctuations [3,10] with low densities over winter, an increase phase in spring, a density peak in late summer, and a decline in autumn (Figure 1b). A non-reproductive winter phase produces two distinct life history patterns: “young-of-the-year” (YY) animals, which breed in the breeding season of their birth (i.e. mature very early, reproduce and die within the same season), and “overwinter” (OW) animals, which are born late in the breeding season, live throughout the winter as subadults (i.e. reproductively inactive but on the basis of their age, able to breed [11]) and mature late in their lives for the next breeding season [12-14]. For OW animals, the life time spent in a premature, non-reproductive state differs among populations and varies with the length of the breeding season, i.e. with latitude or elevation [14-16] and total density [12]. In the high north, with only a short summer and breeding season, all animals have to overwinter before maturation, therefore the population consist always only of OW animals. In more temperate environments, OW and YY cohorts with different life history patterns co-exist during summer (Figure 1a).

Within a temperate environment, age structure across a population should therefore be distinct for each season (Figure 1c): throughout winter, the majority of animals belong to one cohort of subadult OW animals. These animals would be capable of reproducing considering their age, but they are not yet mature [11]. Therefore, when this cohort matures in spring, they are relatively old. During summer, age distribution in a population is bimodal with a few old and multiparous OW animals, and many young YY animals. The age gap between the two modes reflects the length of the non-breeding season (Figure 1c). In autumn, the old OW animals have died, and age structure is no longer bimodal. It is the highest population density as there are several cohorts of YY animals that are reproducing, and young immature OW are born in late breeding season. However, life history tactics for the YY cohort are potentially flexible and may vary inter-annually: if born in the summer of a high-density year, young females may delay their maturation until the next summer, while in low-density years they may reproduce in the summer of their birth [12], thereby creating inter-annual density fluctuations that overlay the intra-annual density fluctuations.

The two modes of life history patterns with their different pace-of-lives (POL) [17] may also relate to different, consistent behavioural patterns. Different POLs among species or breeds can possibly explain their behavioural characteristics [18,19]. According to a model by Wolf *et al*. [20], different POLs may drive the development of animal personalities. Animal personality, a set of correlated behavioural traits, e.g. [21-24], is often characterized by the extremes along a continuum, such as the *shyness – boldness – axis*[25,26]. Wolf et al. (2007) [20] suggest that long-living, late reproducing individuals profit from shy and careful personalities, while short-lived, early reproducing individuals profit from boldness and risk-taking. By applying this model to small mammal populations, we propose that long-lived OW organisms should be shy and careful since inconspicuousness during winter could increase survival chances, while short-lived YY should be bold to increase their competitiveness for finding food and mates during summer [27].

In this study, we compared antipredatory behaviour in a population of common voles across seasons. Common voles display typical annual variation in their population dynamics [28,29]. We further investigated the frequency distribution of behavioural traits within a season to relate it to the distribution of cohorts or to the POL most prevalent at the season. According to the hypothesized connection between POL and animal personality, we expected to find shyer behaviours during winter and spring, when all animals are OW, and a mixture of shy and bold behaviours during summer and autumn when both life history patterns are present.

We propose that for typical prey animals, latency until exposition and activity level are a measure of an individual’s predation risk. Small rodents are prey to a number of avian and terrestrial predators. Hardly any die of old age, and mortality is usually caused by predation [30]. Males, more active and mobile than females in most rodent species, typically face higher predation rates [31,32]. Predator avoidance is therefore an important behaviour that increases the fitness of individuals. Measurable traits like boldness and the activity level of an individual, tested in standard behavioural tests like the open-field, are likely reflecting compromises between the opposing traits of antipredatory behaviour and exploration. Explorativeness may be important in intraspecific interactions, territory defence or in successful foraging.

**Figure 2:**
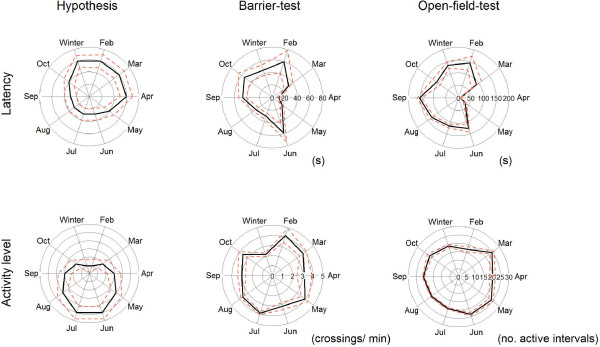
**Hypotheses for behaviour and measured behaviour of 492 common voles around the year.** Voles were captured as adults and tested using two different behavioural tests within a week. Latencies were measured in seconds and activities as frequency of jumps (barrier-test) or as number of active 10 s intervals (open-field). Solid lines indicate means, dotted lines standard error of mean.

We used latencies, measured in standard behavioural tests for anxiety, as a metric for boldness. These latencies are high if the animal is shy and low if it is bold. We further measured activity levels, which should be highest during reproductive season. Our predictions for latencies and activities around the year are visualized in Figure 2.

## Methods

### Animals

492 common voles were captured around Potsdam, Germany (E13°00′ N52°26′) in 2010 (198 individuals), 2011 (69), 2012 (213) and 2013 (12). We used *Ugglan* mice and vole traps (Ugglan special No2, Grahn AB, Sweden) with shrew-exits to avoid the capture of shrews [33]. Traps were baited with rolled oats and apples. We checked traps at least every 12 h. Animals heavier than 15 g and showing scrotal testis or open vagina were considered to be adults in the summer and were removed from the field. Smaller animals and lactating females were released at the point of capture. In winter, all animals were removed.

Captures reflect the population dynamics and sex ratio throughout the different months (Table 1) with higher numbers in summer and autumn than in winter and spring. A female-biased sex ratio during summer, as reflected in our captures (Table 1), is typical for populations of *Microtus* voles [34,35]. This is probably because the mobile males have higher predation rates than the more sedentary females.

**Table 1:** Sample size, study year, month (roman numerals) and sex ratio of common voles captured near Potsdam, Germany

		**Month**												**Sum**
		**I**	**II**	**III**	**VI**	**V**	**VI**	**VII**	**VIII**	**IX**	**X**	**XI**	**XII**	
year	2010	-	-	-	11	15	9	38	39	67	19	-	-	198
	2011	-	5	9	0	5	26	5	-	19	-	-	-	69
	2012	-	-	-	-	-	-	94	54	10	3	52	0	213
	2013	2	10	-	-	-	-	-	-	-	-	-	-	12
sum		2	15	9	11	20	35	137	93	96	22	52	0	492
male/female ratio		-	1.3	0.5	0.6	1.5	0.7	0.5	0.5	1.3	0.6	1	-	0.7
season (n)		winter (17)		spring (40)			summer (265)			autumn (170)			winter	
cohorts expected		middle aged OW		old OW			old OW + young YY			old YY + very young OW				

“ - ” indicates months without sampling.

Voles were kept in standard laboratory mice cages (Ehret GmbH, Germany, Typ III: 42 cm x 27 cm x 16 cm) with water, food pellets (ssniff V1594 R/M-H Ered II), and hay *ad libitum*. The animals were kept on wood shavings with paper rolls for shelter at room temperatures of 18–23°C. After 3–6 weeks of acclimatisation and after pregnant females had given birth and weaned their young, animals were behaviourally tested. After the experiments, the animals either stayed in the laboratory for further experiments (A. Herde et al., unpublished) or were released at their trapping sites.

Animals were captured under permission of the *Landesumweltamt Brandenburg* (reference number RW-7.1 24.01.01.10). Behavioural experiments were conducted under the permission of the *Landesamt für Umwelt, Gesundheit und Verbraucherschutz Brandenburg* (reference number V3-2347-44-2011).

### Behavioural tests

We modified the barrier-test and the open-field-test. These are standard laboratory tests which were originally used to test emotionality or fearfulness in mice and rats; they are now used in studies on animal personality with other species [21]. Latencies and activity levels are behavioural components of conspicuousness of a potential prey animal to a potential predator; they therefore measure an antipredatory component on the boldness-shyness axis. We adjusted the set-ups of the tests for the needs and skills of non-climbing, subterranean, wild-caught voles. Tests were directly observed under conditions similar to the housing room. Observers remained motionless beside the arena and watched the animal either through a Perspex plastic (barrier-test), or directly at the opposite wall and through a mirror (open-field-test) at the wall near the observer to avoid bending over the arena walls. From each test a measure of boldness (i.e. latencies) and a measure of activity level (i.e. crossings per minute / no. of active intervals) were obtained. Both tests were conducted within 2–4 days, but not on the same day and at random order.

For the barrier-test [36,37], a semi-transparent plastic box (45 cm × 22 cm × 25 cm) was divided into two equal compartments by a 4.5 cm high plastic barrier. According to a pseudo-random schedule, the animal was placed in one of the compartments and the latency was measured until the animal crossed the barrier into the other compartment. To obtain an estimate of activity level, the number of crossings within 5 minutes was counted the variable ‘crossings per minute’ (number of crossings adjusted for latency) was calculated.

For the open-field-test [38], we used a round arena (1 m diameter, metal wall 35 cm high) with a 10 cm safe zone along the wall and an unsafe middle zone. The animal was placed in the centre of the arena in a tube. The tube was lifted. All animals sought to leave the centre. The test duration of 5 minutes started at the moment the vole reached the wall of the arena the first time. Latency to enter the zone was measured. In addition, activity levels (all types of movement except fur cleaning) was recorded with instantaneous sampling every 10 seconds, therefore activity levels varied between 0–30 intervals.

### Statistics

Behavioural variables were compared by grouping three months to each season, resulting in 15, 39, 268, and 168 behavioural samples from winter, spring, summer, and autumn, respectively. We used the meteorological year, starting at December 1st, which groups the 3 coldest weeks of the year into winter (in the northern hemisphere) and the warmest into summer, in contrast to the astronomical year (starting Dec 21st). Daylight hours and temperatures for the seasons at the study site during the study period are given in Table 2.

**Table 2:** Meteorological seasons for the study site near Potsdam, Germany

**Season**	**Months**	**Daylight hours**	**Change in daylength**	*Mean T (C°)	*T (min-max) (C°)
Winter	Dec-Feb	7.5 - 10.8	Stable	4.3	−7.5 - 6.5
Spring	Mar-May	10.8 - 16.5	Increasing	15.5	0.2 - 22.1
Summer	June-Aug	13.1 - 16.8	Stable	22.6	11.5 - 29.3
Autumn	Sep-Nov	13.1 - 8.0	Decreasing	15.8	1.3 - 21.1

*T = Temperatures for the years 2010–2012 using data from the German Weather Service (dwd.de) for Potsdam: *Mean T*: mean of 9 monthly (3 months from each of the 3 years) means (arithmetic means of daily mean temperature), minimum and maximum: extreme values of monthly extremes (arithmetic means of daily extremes).

Latencies were log-transformed and activity counts were square-root transformed. We conduced one multivariate Analysis of Variance (MANOVA) for all four variables since each animal was statistically treated as a case with four variables. We used month and sex as factors, calculated with SPSS (Version 20, IBM). MANOVA (if significant) was followed by ANOVAs of the single behavioural variable, and months (if significant) were compared by post-hoc tests. We used Games-Howell for post-hoc analysis, since both sample sizes and variances between seasons were unequal.

## Results

**Figure 3:**
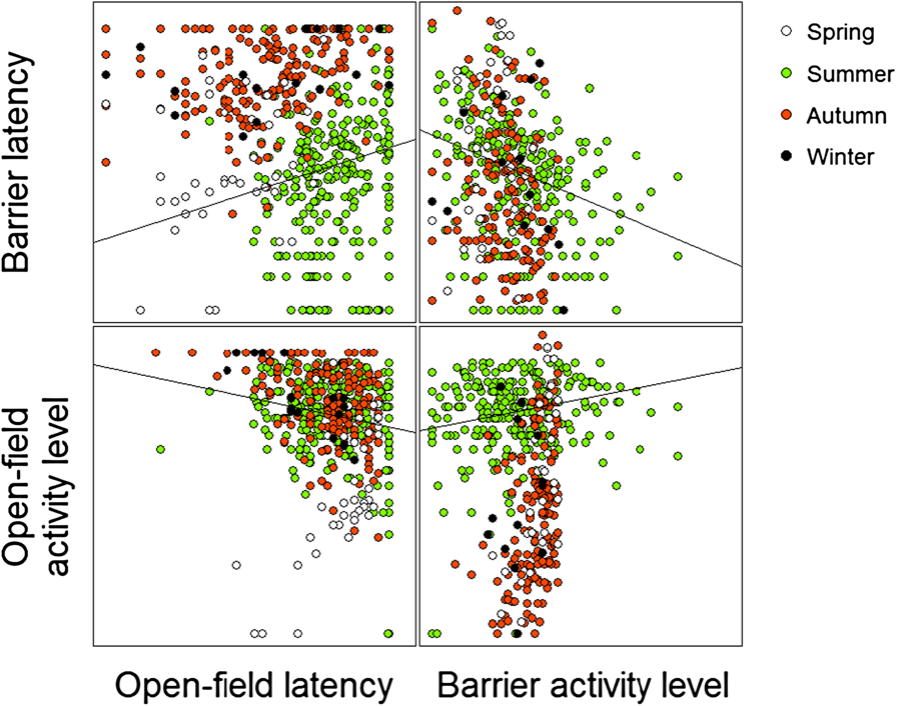
**Correlations of behavioural variables in common voles.** Latency and activity measured in the open-field-test and barrier-test (n = 492 individuals) in different seasons. In the open-field-test we measured both the latency (s) to leave the secure wall of the test arena and activity level (no. of 10 s intervals were animal was active). In the barrier-test we measured the latency (s) until first crossing of barrier into unknown compartment and activity level (crossings per minute).

Mean latencies were 38.8 s (± *SD*, 57.1 s) for the barrier-test and 123.6 ± 90.8 s for the open-field-test. Mean activity level was 3.4 ± 2.5 barrier crossings/minute and 20.5 ± 6.4 active intervals (out of 30 possible) in the open-field. Latencies from both tests were positively correlated with each other (Spearman’s *rho* = 0.24, *p* < 0.001), activity levels from both tests were positively correlated (*rho:* 0.28, *p* < 0.001), and within-tests activity level and latency were negatively correlated (barrier: *rho* = −0.35, *p* < 0.001, open-field: *rho*: -0.27, *p* < 0.001, data and sample size, Figure 3).

Multivariate ANOVA of all four behavioural variables within animal revealed an effect of both sex and season (Table 3) without interaction (removed from the model reported in Table 3 since p in the initial model was F = 0.8, p = 0.554). Season significantly impacted all variables (between subject effects, Table 3), whereas sex only significantly altered the activity level in the open-field.

**Table 3:** **Multivariate ANOVA of behavioural data from two behavioural tests with 492 common voles ( *Microtus arvalis *) pertaining to season of capture and sex of the animal**

**Multivariate test**					
Effect	Wilks-Lambda	F	Hypoth. df	Error df	Sig.
Constant term	.038	2943.8	4	471	.000
Sex	.950	6.2	4	471	.000
Season	.700	15.0	12.0	1246.4	.000
**Between subjects tests**					
Effect	Source	df	MS	F	Sig.
Constant term	Barrier latency	1	260.5	851.8	.000
	Barrier activity level	1	1853.9	302.5	.000
	Open-field latency	1	525.7	3918.0	.000
	Open-field activity level	1	3023.5	4697.0	.000
Sex	Barrier latency	1	0.6	2.0	.157
	Barrier activity level	1	10.7	1.7	.187
	Open-field latency	1	0.2	1.5	.218
	Open-field activity level	1	10.1	15.7	.000
Season	Barrier latency	3	2.4	7.9	.000
	Barrier activity level	3	22.2	3.6	.013
	Open-field latency	3	7.1	52.9	.000
	Open-field activity level	3	2.8	4.4	.005
Error	Barrier latency	474	0.3		
	Barrier activity level	474	6.1		
	Open-field latency	474	0.1		
	Open-field activity level	474	0.6		

The shortest latencies (i.e. boldest behaviour) in the open-field, compared to all other seasons, were measured in spring, and differed from longest latencies in autumn (descriptives for the seasons and post-hoc tests in Table 4). In the yearly cycle, the bolder spring behaviour can clearly be seen in a depression of latencies from March to May (Figure 2). Latencies in barrier-test were not significant different between spring and summer, but were both compared to autumn and winter (Table 4).

**Table 4:** Behavioural variables tested in 492 common voles at 4 seasons

	**Latencies (log(s + 1))**						**Activity levels**					
**Season**	**Open-field**			**Barrier-test**			**Open-field (sqrt(count))**			**Barrier-test (cross/min)**		
	**Mean**	**SD**	**Post-hoc**	**Mean**	**SD**	**Post-hoc**	**Mean**	**SD**	**Post-hoc**	**Mean**	**SD**	**Post-hoc**
Winter	2.08	0.31	b	1.48	0.74	ab	3.90	0.83	a	3.53	2.61	ab
Spring	1.24	0.62	a	1.09	0.45	a	4.75	0.71	b	3.69	1.91	ab
Summer	1.97	0.34	b	1.17	0.59	a	4.43	0.87	ab	3.73	2.03	a
Autumn	2.05	0.34	b	1.42	0.50	b	4.45	0.81	ab	2.87	3.13	b

Descriptives of transformed variables, pairwise post-hoc comparisons (Games-Howell) among seasons**,** compact letter display: different letters indicate significant differences p < 0.05 among months.

Activity levels in the open-field test were highest in spring and were significantly different from the lowest activities levels in winter. Activity levels in the barrier-test were highest in summer, differing from the lowest in autumn (Table 4). Males were more active (22.0 ± 5.9 intervals) than females (19.5 ± 6.5 intervals) in the open-field.

## Discussion

Common voles captured in spring were bolder than animals captured during other seasons. Our predictions for different life history patterns relating to different behavioural traits were not met: we had proposed that OW animals should be more careful, shy, and possibly inactive to survive the winter [27], in accordance to the POL concept [17,19,20]. This should have produced higher test latencies in winter and spring compared to summer (hypothesis in Figure 2). However, as long as OW were subadult (in winter), this was true, but once matured (in spring), we observed the boldest measurements in our sample (Figure 2). In the following, we discuss our results within a theoretical framework of adaptive, age- and cohort-dependent asset-protection, and annual population density cycles. We further discuss possible mechanisms that can produce coherent behaviour within a season and inconsistencies over an individual’s lifetime. We also discuss how our results may affect the application of the concept of animal personalities in seasonal environments.

Higher risk-taking of old OW animals in spring may be adaptive within an asset-protection framework. State and behaviour may interact in a variable environment with several feedback loops [39] Risk-taking and activity can be interpreted as an investment into behaviours that enable animals to reproduce, i.e. finding a mate or securing a rank or a territory. Older animals should display higher investments into reproductive effort than younger animals because of their low residual reproductive value [39,40]. Old, OW animals in our study may therefore have traded-off predator avoidance for competitiveness, thus behaving bolder, while young YY in summer may have invested in survival and predator avoidance rather than competitiveness.

Second, independent of a parent’s life history, offspring born early in a breeding season may have higher fitness value than offspring born late, either because of a longer season to grow larger, or because of an earlier maturation and subsequent longer contribution to the population’s reproduction [41,42]. In short-lived, iteroparous species with annual density fluctuations, the fitness value of an offspring demonstrates extreme changes according to the phase of the annual population cycle. Born at low spring densities into the prospect of increasing summer densities, the value of an offspring and its contribution to the increasing population is larger than that of an offspring born in a high population density with a low chance to contribute to a decreasing population [4]. Therefore, parents should take higher risks in spring to secure their contribution to early offspring, which may have been reflected in the bold behaviour measured in spring.

Behaviour may change with an interaction of life history, personality, state and environment [39] and here we discuss four possible mechanisms that adapt behaviour to the season. First, in many species, hormonal changes can be triggered by seasonal changes in day length [8]. Hormones affecting reproduction often also affect aggression levels [43-45]. Yet if this theory alone explained our results, we would have found boldness in all but the winter sample, since voles reproduce in all seasons except winter. Second, another proximate, physiological explanation for differential behaviours among seasons may be a differential metabolic rate, since animals adapt their metabolism to ambient temperatures and food availability [7], which has possibly changed between winter and spring, but does not explain lower boldness in summer. Third, litter size of the animals tested as adults may provide a mechanism of connecting behaviour to life history by the *sibling effect*: the number of siblings (i.e. litter size) an individual experiences during its ontogeny can affect its behaviour later as an adult [46]. In vole populations, old OW females produce small first litters in spring and larger second and third litters in summer, while YY females produce larger first litters [47]. Even in laboratory colonies of voles without a seasonally different environments, older females’ first litters were smaller than their second litter, while extremely young females had large first litters [48]. This age-dependency of litter size, combined with the typical annual age structure of a vole population (Figure 1) results in population-wide smaller litters in spring (when YY animals are born) and large litters in summer and autumn (when OW are born) [27]. The behaviour of the cohort emerging from these litters may thus be adapted to the seasonal population cycle via the sibling effect [27]. Fourth, prey animals can adapt their antipredatory behaviour to seasonal differences in predation pressure [49]. It is conceivable that the predation pressure during summer is higher since all predators have to feed their young. On the other hand, vole numbers have increased so that per-capita predation rate may actually be constant. Unfortunately, we do not have estimates of predation pressure for the populations our test animals were captured from.

Behaviour of individuals was correlated between the two tests, indicating that animals that behaved bold in one test also do so in the second. In a parallel study on voles kept in the laboratory, we found that individual differences in behaviour of common voles tested repeatedly were rather consistent over time, even over maturation (A. Herde & J.A. Eccard, unpublished). Both results indicate the existence of animal personalities in this species as well. However, results on animal personality often refer to a group level phenomenon, i.e. the same animals rank similarly at different points in time [50]. Since we have not captured the same group of animals twice we cannot test whether the same animals would rank similarly in different seasons. Nevertheless, our study shows that environmental conditions, food availability and population level changes in physiology may largely determine the parameter space in which an entire population behaves, i.e. the phenotypic plasticity of behaviour population wide [50]. This needs to be understood as a background when applying the concept of animal personality studies onto short-lived animals in seasonal environments. However, relating life-history or POL of different cohorts to the behaviour of these cohorts using their absolute trait values is apparently difficult in short-lived animals *in situ*, since cohorts hardly overlap (Figure 1). Even when they overlap during their lifetime, as in summer (Figure 1), they will never have the same age. We still suggest long time capture-mark-recapture (CMR) studies of voles with repeated, behavioural measurements taken to disentangle effects of individual behaviour, seasonality, phenotypic plasticity and animal personalities.

Activity levels of common vole males were higher than those of females. Population genetics suggest that male common voles move among population clusters since females mate promiscuously [51], while females are philopatric and live in kin-clusters [52]. Females may wait to be visited by males and do not increase their mobility for finding a mating partner, similar to many other rodents [53]. This adaptation to voles’ breeding systems may explain the measured differences in behaviour between the sexes. Higher activity levels of males may explain their higher mortality rates [31,32] and female-biased sex ratios in our samples (Table 2).

## Conclusion

Complex patterns of population dynamics and seasonal variation in life histories may cause complex behavioural dynamics in a population. Our observation suggests different behavioural trait values among seasons, but also consistencies of behavioural traits over time in voles (A. Herde & J.A. Eccard, unpublished). To understand individual variation, plasticity and seasonality we suggest studies on individual behaviour in a population *in situ,* covering the entire life span of a vole.

## Abbreviations

OW: Overwinter; YY: Young-of-the-year; POL: Pace-of-life; BMR: Basal metabolic rate; MANOVA: Multivariate analysis of variance; ANOVA: Analysis of variance; CMR: Capture-mark-recapture.

## Competing interests

The authors declare that they have no competing interests.

## Authors’ contributions

JAE and AH designed the study. AH performed the data collection. JAE analysed the data and wrote the paper, both authors revised the paper. Both authors read and approved the final manuscript.
